# Proteomic identification of mammalian cell surface derived glycosylphosphatidylinositol-anchored proteins through selective glycan enrichment

**DOI:** 10.1002/pmic.201400148

**Published:** 2014-11-12

**Authors:** Leslie K Cortes, Saulius Vainauskas, Nan Dai, Colleen M McClung, Manesh Shah, Jack S Benner, Ivan R Corrêa, Nathan C VerBerkmoes, Christopher H Taron

**Affiliations:** New England Biolabs, IncIpswich, MA, USA

**Keywords:** Alkyne agarose, Azido sugar analog, Glycoproteins, Lectin affinity, Polarized epithelial cells

## Abstract

Glycosylphosphatidylinositol-anchored proteins (GPI-APs) are an important class of glycoproteins that are tethered to the surface of mammalian cells via the lipid GPI. GPI-APs have been implicated in many important cellular functions including cell adhesion, cell signaling, and immune regulation. Proteomic identification of mammalian GPI-APs en masse has been limited technically by poor sensitivity for these low abundance proteins and the use of methods that destroy cell integrity. Here, we present methodology that permits identification of GPI-APs liberated directly from the surface of intact mammalian cells through exploitation of their appended glycans to enrich for these proteins ahead of LC-MS/MS analyses. We validate our approach in HeLa cells, identifying a greater number of GPI-APs from intact cells than has been previously identified from isolated HeLa membranes and a lipid raft preparation. We further apply our approach to define the cohort of endogenous GPI-APs that populate the distinct apical and basolateral membrane surfaces of polarized epithelial cell monolayers. Our approach provides a new method to achieve greater sensitivity in the identification of low abundance GPI-APs from the surface of live cells and the nondestructive nature of the method provides new opportunities for the temporal or spatial analysis of cellular GPI-AP expression and dynamics.

## 1 Introduction

**Figure 1:**
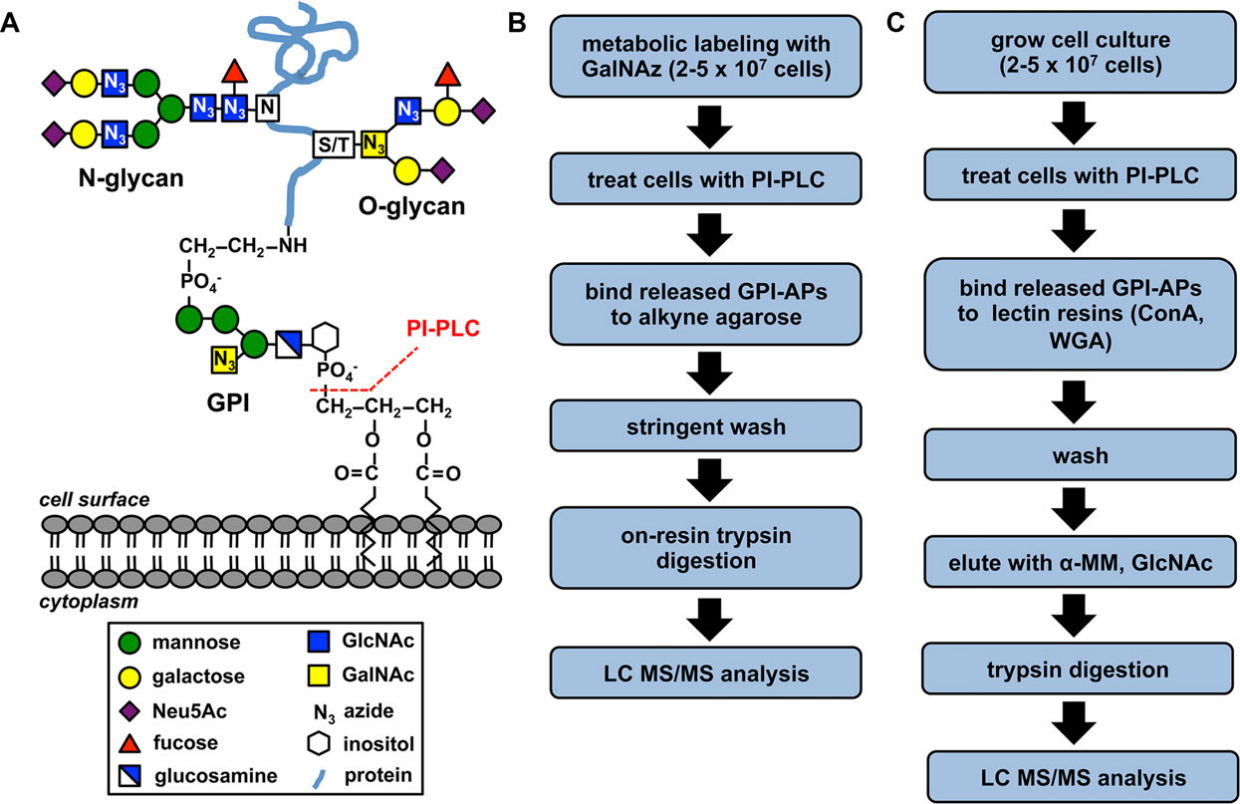
Schematic of a representative GPI-AP and experimental workflows. (A) Schematic of a representative GPI-AP. The sugar molecules of the GPI anchor, a representative N-glycan appended to asparagine (N) and a representative O-glycan attached to serine or threonine (S/T) are shown. Sites of potential GalNAz incorporation into GPI-AP glycans are indicated with an N_3_ in the sugar symbol. The site of PI-PLC cleavage resulting in release of the GPI-AP from the cell surface is also indicated. (B) Workflow for the sugar analog capture enrichment. (C) Workflow for the lectin affinity capture enrichment. Neu5Ac: *N*-acetylneuraminic acid; GlcNAc: *N*-acetylglucosamine; GalNAc: *N*-acetylgalactosamine; PI-PLC: phosphatidylinositol-specific phospholipase C; GalNAz: *N*-azidoacetylgalactosamine; WGA: wheat germ agglutinin; α-MM: methyl α-d-mannopyranoside.

Glycosylphosphatidylinositol-anchored proteins (GPI-APs) are a class of proteins tethered to the plasma membrane of cells that perform or mediate a variety of critical cellular functions including signal transduction, immune recognition, complement regulation, and cell adhesion. The GPI anchor consists of a conserved core glycan linked on its reducing end to the lipid phosphatidylinositol and covalently attached to protein via phosphoethanolamine on its nonreducing end (Fig. 1A). GPIs are assembled and transferred *en bloc* to the C-termini of various secretory glycoproteins in the ER. GPI-APs are then transported to the cell surface via the secretory pathway. In addition to its GPI anchor, most characterized GPI-APs also possess additional carbohydrate modifications such as N- and/or O-linked glycans (Fig. 1A) 1–4.

GPI anchors are essential for the correct cell surface localization and function of their appended proteins (for reviews see 5–7). While mammalian cells can survive in vitro without GPI anchoring, a complete loss of GPI anchor biosynthesis is embryonic lethal during mammalian development 8 and a clonal loss of GPI anchoring results in the acquired hemolytic disease paroxysmal nocturnal hemoglobinuria 9. Furthermore, cell surface GPI-APs serve as important biomarkers in cellular differentiation and disease. For example, the diagnosis of paroxysmal nocturnal hemoglobinuria involves measuring decreased surface expression of GPI-APs 10. Additionally, increased expression of certain GPI-APs has been observed in various types of cancer, with some being used as prognostic indicators 11–13. Finally, GPI-APs such as CD73, CD106, Sca-1, and CD90 are important stromal cell-associated markers that have been used for the identification of mesenchymal stem cells 14. Thus, the ability to identify the cohort of GPI-APs present on the surface of cells may uncover novel markers for cell differentiation and disease.

Polarized epithelial cells contain discrete apical (AP) and basolateral (BL) plasma membrane domains that have unique protein and lipid compositions. Many studies have reported preferential localization of GPI-APs to the apical membrane 15–20. Additionally, correct membrane localization of certain GPI-APs has been shown to be critical for specific processes such as viral infection 21 and cell signaling 22. Since the first report of apical trafficking of GPI-APs almost 25 years ago 23, the mechanism of polarized localization of GPI-APs has been the subject of much work and debate (see 24 for a review). Many of these studies used heterologous GPI-anchored reporter proteins due to low expression of endogenous GPI-APs and challenges in visualizing specific GPI-APs on the surface of live cells. However, little is known about the population of endogenous GPI-APs present in apical and BL membranes. Defining the cohort of endogenous GPI-APs within each membrane domain using proteomics could ultimately contribute to a better understanding of their trafficking.

Computational prediction of GPI-APs from genomic sequence information has suggested that the number of GPI-APs encoded by the human genome is potentially in the hundreds (this study and 25,26). However, experimentally, the human GPI proteome is still poorly defined. One factor that complicates proteomic analyses of mammalian GPI-APs is their low abundance compared to other cell surface proteins. Thus, prior GPI-AP proteomic studies employed enrichment methods to increase the density of GPI-APs in a sample. Studies in HeLa cells used the detergent-based two-phase partitioning of membrane proteins to concentrate GPI-APs followed by enzymatic release of GPI-APs from these membranes with phosphatidylinositol-specific phospholipase C (PI-PLC) or GPI-specific phospholipase D 27,28. More recently, a study investigating the GPI-APs present in membranes isolated from breast cancer cells used the binding of PI-PLC-released GPI-APs to bacterial alpha-toxin, a protein that binds specifically to the glycan core of the GPI anchor, as a means to further enrich samples for GPI-APs prior to MS analysis 29. Each of these GPI-AP enrichment strategies had limited success in identifying GPI-APs. Other studies addressing the whole plasma membrane proteome or the proteome of lipid rafts also identified some GPI-APs 30–33. Importantly, all of these studies used sample preparation methods that destroyed the cells being analyzed thus eliminating the ability to identify mature GPI-APs that dynamically populate the surface of live cells.

In the present study, we report methodology that permits the identification of GPI-APs en masse directly from the surface of intact mammalian cells. Our approach uses either of two methods that selectively capture and concentrate GPI-APs enzymatically released from the surface via their ubiquitous appended carbohydrates (N-linked, O-linked, and GPI glycans) followed by LC-MS/MS analysis. In the first enrichment scheme, in vivo metabolic labeling of GPI-APs with an azido sugar analog was performed and PI-PLC-released proteins were enriched via capture on alkyne agarose resin. The second enrichment scheme used lectins to capture and enrich for PI-PLC-released GPI-APs. Using these approaches, we performed analysis of three different mammalian cell lines and demonstrated a significant increase in sensitivity of mammalian GPI-AP identification. Furthermore, our ability to identify GPI-APs without first disrupting cellular structure permitted us to separately identify GPI-APs present in discrete membrane domains (apical and BL surfaces) of polarized epithelial cells. Our study advances the use of proteomic methods to define the mammalian GPI-AP proteome and should further enable discovery of novel GPI-AP biomarkers associated with cellular differentiation or disease.

## 2 Materials and methods

### 2.1 Chemical synthesis of tetraacetylated *N*-azidoacetylgalactosamine (GalNAz) and reconstitution

Per-*O*-acetylated GalNAz was prepared in four steps and 59% overall yield according to a protocol described by Laughlin and Bertozzi 34. In short, bromoacetic acid (Sigma-Aldrich) was converted into azidoacetic acid *N*-succinimidyl ester and subsequently reacted with galactosamine hydrochloride (Carbosynth). The resulting GalNAz was peracetylated in the presence of acetic acid and pyridine, and purified by flash chromatography, eluting with 7:3 hexanes/ethyl acetate. GalNAz was resuspended in 100% ethanol for a 50 mM stock solution.

### 2.2 Cell culture and GalNAz labeling of mammalian cells

HeLa cells (ATCC) were cultured in DMEM (Thermo Scientific HyClone) containing 10% v/v FBS (Thermo Scientific HyClone), 2 mM l-glutamine, 100 U/mL penicillin, and 100 μg/mL streptomycin. Madin–Darby canine kidney cells (MDCK; Type II) cells (Sigma) were cultured as nonpolarized or polarized monolayers in MEM/EBSS (Thermo Scientific HyClone) containing 5% v/v FBS, 2 mM l-glutamine, 100 U/mL penicillin, and 100 μg/mL streptomycin. Polarized monolayers were grown for 4 days on 75 mm diameter, 0.4 μm pore size polycarbonate membrane Transwell supports (Corning) seeded at a density of 1 × 10^7^ cells/cm^2^ with daily medium changes. Human retinal pigment epithelium cells (ARPE-19; ATCC) were cultured as nonpolarized cells in DMEM/Ham's F12 containing 10% v/v FBS, 2 mM l-glutamine, 100 U/mL penicillin, and 100 μg/mL streptomycin. For the growth of polarized monolayers, cells were seeded at a density of 1.6 × 10^5^ cells/cm^2^ on Transwell supports. The serum in the medium was reduced to 1% v/v 48 h after plating and cells were maintained for 3–4 weeks prior to use. For GalNAz labeling, 200 μM GalNAz was included in the cell medium 48–72 h prior to PI-PLC release of GPI-APs. The integrity of the polarized monolayer was verified by setting up medium disequilibrium and ensuring the media did not equilibrate over a 16- to 24-h period 36.

### 2.3 PI-PLC release of GPI-APs

HeLa (∼5.4 × 10^7^–1.1 × 10^8^), MDCK (∼5 × 10^7^), or ARPE-19 (∼1.1 × 10^7^) cells were washed with DMEM without FBS. Cells were then treated with *Bacillus cereus* PI-PLC 35 (3 units/mL final concentration) or mock PI-PLC treated for 30 min at 37°C in fresh DMEM without FBS. Spent culture medium was harvested after PI-PLC and mock treatments and centrifuged for 10 min at 3000 × *g* to remove any cellular debris. The supernatant was concentrated using a VivaSpin20 concentrator (10 kDa MWCO, Sartorius).

### 2.4 Alkyne agarose purification of GalNAz-labeled proteins

Purification of GalNAz-labeled proteins was performed using a ClickIT Protein Enrichment Kit (Invitrogen) per manufacturer's instructions. On-bead tryptic digestion and LC-MS/MS analysis are described below. Biological triplicate samples were prepared and analyzed for all sugar analog enrichment (SAE) experiments.

### 2.5 Enrichment of GPI-APs using lectin resins

Following concentration of supernatant, 250 μL of 5× binding/wash buffer (100 mM Tris-HCl pH 7.5, 2.5 M NaCl, 25 mM MgCl_2_, 25 mM MnCl_2_, and 25 mM CaCl_2_) was added to the supernatant, and the sample was bound for 30 min to a column containing 50 μL of each Concanavalin A (ConA) and wheat germ agglutinin (WGA) lectin resins (Sigma) preequilibrated with 1× binding/wash buffer. The resin was washed with 5 mL of 1× binding/wash buffer, and bound proteins eluted with 250 μL of binding per wash buffer containing 400 mM methyl α-d-mannopyranoside and 200 mM *N*-acetylglucosamine (GlcNAc). Eluted proteins were concentrated using a Microcon spin concentrator YM-10 (Millipore), reduced with 10 mM DTT (70°C, 15 min) and alkylated in a buffer containing 60 mM iodoacetamide (30 min, room temperature). The protein samples were washed in Microcon YM-10 spin concentrators with 3 × 200 μL of 20% ACN v/v and then the buffer was exchanged with 0.1 M Tris pH 8.0, 2 mM CaCl_2_, 10% ACN. Samples were concentrated using spin concentrators to a final volume of 25 μL. Trypsin digestion and LC-MS/MS analysis are described below. Biological triplicate samples were prepared and analyzed for all lectin affinity enrichment experiments.

### 2.6 LC-MS/MS

Solution or bead-immobilized samples were digested overnight with 25 ng/μL trypsin (Promega) at 37°C. Peptides were cleaned and separated from beads using a C_18_ ZipTip (Millipore), concentrated to 10 μL using a SpeedVac, and analyzed by positive ion Top 7 data-dependent acquisition mode LC-MS/MS using a linear ion trap mass spectrometer (LTQ, Thermo Fisher Scientific). Peptides were delivered and separated using an EASY-nLC nanoflow HPLC (Thermo Fisher Scientific) at 300 nL/min using a 75-mm inner diameter × 15-cm length Picofrit capillary column (New Objective) self-packed with 5 mm Magic C18 resin (Michrom Bioresources). Solvent gradient conditions were 60 min from 3% B buffer to 38% B (B buffer: 100% ACN; A buffer: 0.1% formic acid/99.9% water). MS/MS spectra acquired by CID were analyzed using the SEQUEST algorithm (version 27, rev.14) by searching the reversed and concatenated Swiss-Prot protein database (SwissProt_2012_01, containing 1 068 484 sequences across all taxa, http://www.ebi.ac.uk/uniprot/database/) with a parent ion tolerance of 2.0 Da and fragment ion tolerance of 0.80 Da. Carbamidomethylation of Cys (+57.0293 Da) was specified in SEQUEST as a fixed modification and oxidation of Met (+15.9949). The maximum number of missed cleavages permitted was 5. Results were imported into Scaffold 3.3 software (Proteome Software) and probability was adjusted to a 1.2% peptide false discovery rate (FDR) and 0.2% protein FDR. Known contaminants such as keratins, caseins, trypsin, and BSA were removed from the analysis. Full SEQUEST configuration information is available in Supporting Information Table 1. All available MS data for these samples are available at http://proteomicsdata.neb.com/publications/GPIProteomics/.

### 2.7 2D LC-MS/MS

Bead-based samples were digested overnight with 50 ng/μL Trypsin Ultra Mass Spectrometry Grade (New England Biolabs) at 37°C. Beads were spun to the bottom of the tube and only the supernatant was removed, aliquoted, and stored at −80°C until further analysis.

After sample preparation, individual aliquots of the complex peptide mixture were loaded onto a split phase 2D RP-strong cation exchange (SCX) back column. The SCX phase was 150 μm × ∼3–5 cm (Luna SCX, 5 μm particle size, 100 Å pore size, Phenomenex, CA, USA) and the reverse phase was 150 μm × ∼3–5 cm (AQUA C18, 3 μm particle size, 300 Å pore size, Phenomenex). Column was packed using a PicoView Pressure Injection Cell (New Objective). After loading, the RP-SCX column was connected to the HPLC and washed with 100% aqueous solvent for 5 min and then ramped up to 100% organic solvent (70% ACN, 0.1% formic acid) over 10 min. This migrates peptides from the RP phase onto the SCX phase and effectively desalts the peptide samples and removes other nonpeptide contaminants which do not bind to the SCX. The back column was then connected to a 100 μm × 15 cm RP resolving front column with an integrated Nanospray tip (AQUA C18, 3 μm particle size, 300 Å pore size, Phenomenex) resting on the Proxeon Nanospray source (Proxeon Biosystems, Odense, Denmark) attached to a Q Exactive mass spectrometer (Thermo Fisher Scientific). An automated 2D LC-MS/MS run was programed into Xcalibur (Thermo Fisher Scientific) and each sample was analyzed with a three-salt step followed by 2 h C18 separation for a total of 6 h analyses time per sample 37. During the entire 2D LC-MS/MS analyses, the Q Exactive operated in data-dependent mode with top ten MS/MS spectra (one microscan, 17 500 resolution) for every full scan (one microscan, 70 000 resolution). Dynamic exclusion was turned on with a 15-s interval and normalized collision energy was set at 28.0%.

RAW files from each 6 h 2D LC-MS/MS analysis were extracted into mzXML files, using the MSConvert utility from ProteoWizard suite of tools (http://proteowizard.sourceforge.net). The search database was constructed using a recent canine-predicted protein database (NCBI DogRefSeq, CanFam3.1, September 2013 assembly, containing 34 594 proteins), the common contaminants (trypsin, keratin, etc.), and lab protein standards (BSA, hemoglobin, etc.). Searches were done with the MyriMatch search engine (Version 2.1.138) 38. The parent ion tolerance of 20 ppm and the fragment ion tolerance of 30 ppm were specified. Carbamidomethylation of Cys (+57.0293 Da) was specified as a fixed modification. The maximum number of missed cleavages was set to 2. Resulting pepXML output files were analyzed with IDPicker 39 for assembling the raw peptide identifications from MyriMatch into confident protein identifications. FDR for each sample run was calculated by IDPicker based on the reverse database target-decoy search strategy 40 with a maximum FDR parameter set to 2%. Calculated FDRs were <1.35% across all sample runs. All MyriMatch configuration information is available in Supporting Information Table 1. The MS proteomics data have been deposited to the ProteomeXchange Consortium (http://proteomecentral.proteomexchange.org) via the PRIDE partner repository 41 with the dataset identifier <PXD001130>. Also all data from this analysis are publically available via http://proteomicsdata.neb.com/publications/GPIProteomics/.

### 2.8 Analysis of MS data

For each list of proteins from the LC-MS/MS analyses, candidate GPI-APs were identified either through annotation in the UniProt database, prediction of a GPI attachment site by FragAnchor and/or PredGPI, or prior experimental evidence of a GPI anchor. Intracellular and multipass transmembrane proteins were eliminated from the analysis. In cases where multiple splice forms of a protein exist and only a subset of those splice forms were predicted to be GPI anchored, the peptide spectra were analyzed to ensure they matched the GPI-anchored splice form(s) of the candidate protein. A protein had to contain at least two unique peptide matches and two spectral counts in the PI-PLC-released fraction to be included in the list of identified GPI-APs.

## 3 Results

### 3.1 Glycosylation of mammalian GPI-APs

Prior reports have determined that several mammalian GPI-APs each possess experimentally verified combinations of N- and/or O-linked glycans 1–4, however, the potential to which all GPI-APs may receive multiple types of glycosylation has not been systematically examined. Therefore, we computationally modeled and evaluated the human proteome for the presence of GPI-APs and their putative N- and O-linked glycan sites (Supporting Information Table 2). The programs FragAnchor and PredGPI were each used to predict the presence of a C-terminal GPI attachment site (ω site) in all UniProt human proteins 25,26. The data were narrowed to include only proteins having an N-terminal secretion signal peptide (SignalP 4.1) 42, and checked by TMHMM 2.0 43 to ensure proteins contained two or fewer putative transmembrane segments. While GPI-APs do not contain transmembrane domains, prediction algorithms occasionally interpret the N-terminal signal peptide and/or the C-terminal GPI signal sequence as transmembrane helices. The resulting list of 255 human candidate GPI-APs is presented in Supporting Information Table 3. The modeled human GPI-AP dataset was further evaluated for the presence of potential N- and/or O-linked glycan attachment sites using the programs NetNGlyc 1.0 and NetOGlyc 4.0, respectively 44. Nearly all (99%) of the modeled GPI-APs contained predicted N- or O-linked glycan sites (Supporting Information Tables 2, 3). Most of the proteins (92%) possessed putative N-glycans sites with 85% having potential O-glycan sites, and 77% having both. While computational modeling alone cannot ensure that a protein will definitively possess N- or O-linked glycans, this analysis suggests that there is a very high potential for almost all human GPI-APs to harbor multiple forms of appended glycans (N- or O-linked and the GPI glycan).

### 3.2 Enrichment of GPI-APs from complex protein mixtures

Based on the likelihood that the majority of human GPI-APs contain N- and/or O-glycans in addition to their GPI anchors, we sought to use glycans as “handles” to isolate GPI-APs from complex protein mixtures. In contrast to prior GPI-AP proteomic analyses that utilized concentrated membranes from lysed cells, we sought to identify GPI-APs liberated directly from the surface of intact live cells.

Two workflows were established for the purification of GPI-APs from live cells via their appended glycans (Fig. 1B and C). The first method (sugar analog capture enrichment) consisted of metabolically incorporating the azido sugar analog GalNAz into cellular glycans prior to the PI-PLC-mediated release of GPI-APs (Fig. 1B). Previous studies had demonstrated that GalNAz (and its epimerized *N*-azidoacetylglucosamine form) becomes incorporated into N- and O-linked glycans as well as into some GPI anchors 35,45,46. Hence, GPI-APs from labeled cells may contain the sugar analog in multiple glycans (Fig. 1A). Following labeling, click chemistry is used to covalently immobilize GPI-APs to an alkyne agarose resin via their azide-labeled glycans. The captured proteins are then stringently washed and subjected to on-resin trypsin digestion and liberated peptides are analyzed by LC-MS/MS.

The second method (lectin affinity capture enrichment) involves using immobilized lectins to capture PI-PLC-released GPI-APs via their appended glycans (Fig. 1C). In this strategy, which is not dependent on metabolic labeling, PI-PLC-released GPI-APs are bound to a mixture of ConA and WGA lectin resins. ConA binds to α-d-glucose and α-d-mannose containing oligosaccharides, while WGA is thought to bind GlcNAc and sialic acid residues, common substituents of GPIs, N-, and O-linked glycans. The noncovalent interaction between captured GPI-APs and resin allows for competitive elution of bound proteins with α-methyl-mannopyranoside and GlcNAc. Eluted proteins are treated with trypsin and resulting peptides analyzed by LC-MS/MS.

### 3.3 The cell surface GPI-AP proteome of HeLa cells

To determine the reproducibility and effectiveness of our experimental workflows, GPI-APs from HeLa cells were isolated and captured using the sugar analog or lectin enrichment methods. Following trypsin digestion and LC-MS/MS, GPI-APs were extracted from the list of all identified proteins using the methods described in Section 2.8. Of the 33 identified GPI-APs, 27 proteins (82%) were annotated in the UniProt database as GPI-APs. The remaining six proteins have been either experimentally demonstrated to be a GPI-AP (but not annotated as such) or the identified peptides mapped to a protein isoform predicted by one or more algorithms to potentially be GPI anchored (Table1, Supporting Information Tables 4, 5). Control experiments on HeLa cells using PI-PLC treatment alone without sugar analog or lectin affinity enrichment failed to identify as many GPI-APs. There was significant overlap in the GPI-APs identified by both glycan enrichment methods with 16 of 33 proteins (48%) being commonly observed (Table1). The protein identifications were reproducible, with 81% present in two or more biological replicates using sugar analog capture and 78% using lectin capture (Table1, Supporting Information Fig. 1). Importantly, our method identified ten of the 11 GPI-APs previously observed in HeLa cells using PI-PLC and GPI-PLC release on concentrated detergent-resistant membrane preparations 27,28. Consistent with previous reports, some release of GPI-APs in the non-PLC-treated control samples was observed 47–50. However, treatment with PI-PLC enriched our samples for GPI-APs at least threefold over non-PLC-treated controls (Supporting Information Table 6). These data demonstrate that glycan-based enrichment ahead of LC-MS/MS is a viable approach for identification of cell surface GPI-APs. The described workflow is more sensitive than prior methods and can be executed without disruption of cellular membranes.

**Table 1:** Comparison of biological triplicate samples of HeLa cells using sugar analog (SAE) and lectin enrichment (LE)

No.	Identified proteins	UniProtKB	MWa)	Sugar analog		Lectin capture		Summary	
				Avg % Seq Cov (Avg Pepb))		Avg % Seq Cov (Avg Pepb))		SAE	LE
				−PLC	+PLC	−PLC	+PLC	+PLC	+PLC
1	CD109 antigen	Q6YHK3	162	3.2 (4)	13 (16)	3.6 (4)	16.1 (17)	3/3	3/3
2	Folate receptor alpha	P15328	30	4.3 (1)	35.3 (8)	7.1 (1)	27.8 (6)	3/3	3/3
3	Alkaline phosphatase, tissue-nonspecific isozyme	P05186	57	6.3 (2)	28.9 (12)	11.5 (4)	39.6 (16)	3/3	3/3
4	Complement decay-accelerating factor	P08174	41	4.5 (1)	23.8 (8)	5.5 (1)	28.9 (9)	3/3	3/3
5	Glypican-1	P35052	62	14.6 (6)	22.5 (9)	0	0	3/3	0/3
6	Melanotransferrin	P08582	80	0	13.6 (8)	1 (1)	6.3 (5)	3/3	3/3
7	Urokinase plasminogen activator surface receptor	Q03405	37	4.9 (1)	19.7 (4)	3.3 (1)	16.6 (4)	3/3	3/3
8	CD59 glycoprotein	P13987	14	0	14.6 (2)	12.0 (1)	19.3 (3)	2/3	3/3
9	Carboxypeptidase M	P14384	51	0	5.2 (2)	0	2.8 (1)	2/3	1/3
10	Glypican-5	P78333	64	2.9 (1)	3.5 (2)	0	0	2/3	0/3
11	5'-Nucleotidase	P21589	63	0	3.7 (2)	6.2 (2)	7.9 (3)	2/3	1/3
12	Lymphocyte function-associated antigen 3	P19256	28	0	2.4 (1)	0	6.0 (2)	1/3	2/3
13	Cadherin-13	P55290	78	0	4.2 (3)	0	5.1 (3)	3/3	3/3
14	Mesothelin	Q13421	69	4.4 (3)	6.4 (3)	0	1.8 (1)	3/3	1/3
15	NKG2D ligand 3	Q9BZM4	28	0	9.8 (2)	0	0	3/3	0/3
16	Growth arrest-specific protein 1	P54826	36	0	7.3 (2)	0	0	2/3	0/3
17	Testisin	Q9Y6M0	35	0	5.6 (1)	0	0	2/3	0/3
18	NKG2D ligand 2	Q9BZM5	27	2.7 (1)	7.0 (2)	0	2.4 (1)	2/3	1/3
19	Major prion protein	P04156	28	0	5.0 (1)	0	0	2/3	0/3
20	Ephrin-A1	P20827	24	0	0	0	6.7 (1)	0/3	2/3
21	Reticulon-4 receptor-like 2	Q86UN3	46	0	0	0	4.3 (2)	0/3	2/3
22	Ly6/PLAUR domain-containing protein 3	O95274	36	0	0	2.0 (1)	6.8 (2)	0/3	2/3
23	Reticulon-4 receptor	Q9BZR6	51	0	0	2.5 (1)	4.9 (1)	0/3	2/3
24	Laminin subunit alpha-4c)	Q16363	203	12.1 (17)	5.2 (7)	0	0.5 (1)	3/3	1/3
25	Voltage-gated Ca^2+^ channel subunit α-2/δ-1d)	P54289	125	0	3.8 (4)	0	3.1 (3)	3/3	2/3
26	Fibulin-1c)	P23142	77	9.7 (5)	6.5 (3)	3.9 (2)	2.4 (1)	3/3	1/3
27	Fibulin-3c)	Q12805	55	7.1 (3)	0	3.5 (1)	4.6 (2)	0/3	2/3
28	Neuronal growth regulator 1	Q7Z3B1	39	0	0	0	12.3 (3)	0/3	3/3
29	Glypican-4	O75487	62	0	2.0 (1)	0	0	1/3	0/3
30	Ly6/PLAUR domain-containing protein 6B	Q8NI32	21	0	4.9 (1)	0	0	1/3	0/3
31	Hyaluronidase-2	Q12891	54	0	0	0	2.3 (1)	0/3	1/3
32	Intercellular adhesion molecule 5c)	Q9UMF0	97	0	1.1 (1)	0	0	1/3	0/3
33	Deoxyribonuclease-1-like 1c)	P49184	34	0	3.9 (1)	0	0	1/3	0/3

For each enrichment method, the average percent sequence coverage (Avg % Seq Cov) and average number of unique peptides (Avg Pep) across three biological replicates are indicated. The far right columns show the reproducibility of a protein identification using a given enrichment method (+PLC only) across three biological replicates. PLC, phospholipase C.

a) Theoretical molecular weight of precursor protein in kDa.

b) The minimum number of unique peptides required for a protein identification was 2, but due to averaging across triplicate samples, the average peptides may appear to be 1. A complete listing of the number of peptides for each replicate can be found in Supporting Information Table 5 and all peptides used in identifications are listed in Supporting Information Table 6.

c) Peptides match specific isoforms predicted to contain a GPI anchor though other isoforms cannot be excluded.

d) Experimentally proven to be GPI anchored in other studies but not annotated as a GPI-AP in the UniProt database.

### 3.4 GPI proteomics of polarized cells

To highlight the utility of our approach for isolation of cell surface GPI-APs from intact cells, we applied the SAE method to the discrete apical and BL membrane domains of polarized epithelial cells. GPI-APs have been shown to preferentially localize on the apical surface of polarized MDCK and ARPE-19 15–18,20,35,51. However, most of these studies employed overexpressed recombinant GPI-anchored reporter proteins to determine GPI-AP localization. Meanwhile, reports examining the distribution of some endogenous GPI-APs have reported significant levels of GPI-APs on both the apical and BL surfaces 35,47. To obtain a more complete picture of the distribution of endogenous GPI-APs in polarized cells, we examined the apical and BL GPI proteomes of polarized epithelial cell monolayers.

**Figure 2:**
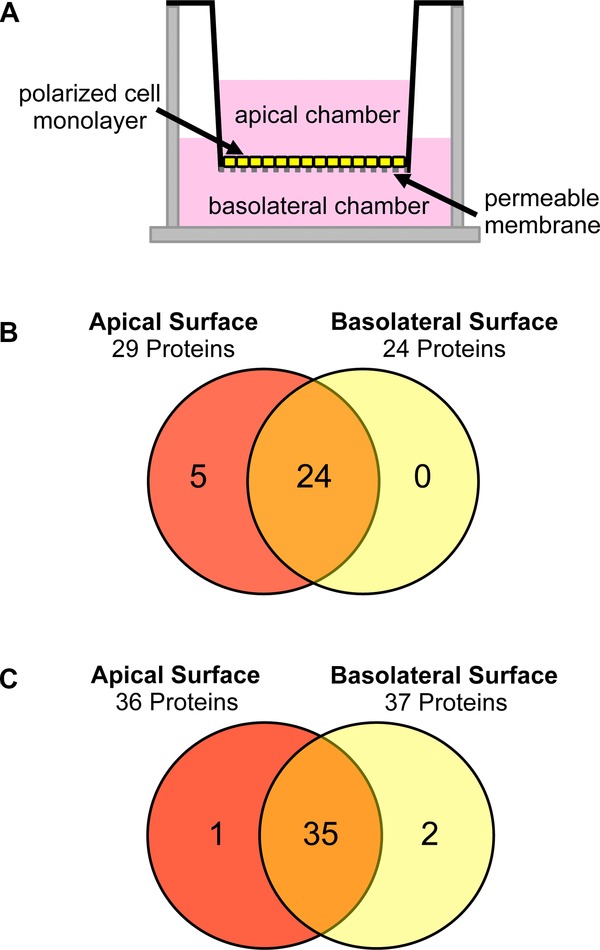
Membrane distribution of GPI-APs from polarized ARPE-19 and MDCK cells. (A) Schematic of a polarized cell culture in Transwell inserts. The cells sit on top of the polycarbonate membrane and once fully polarized, there is no mixing between the apical and basolateral compartments. (B) Venn diagram summary of the results of MS identification of GPI-APs from the apical and basolateral surfaces of polarized ARPE-19 cells. (C) Venn diagram summary of the analysis of polarized MDCK cells.

In a prior study, we established polarized ARPE-19 cells as a model system for in vivo incorporation of GalNAz into GPI anchors and N-glycans 35. Thus, we used GalNAz-labeled ARPE-19 cells to concurrently determine the apical and BL GPI proteomes of a polarized monolayer. In this experiment, ARPE-19 cells were grown as a polarized monolayer on a permeable membrane and labeled with GalNAz (Fig. 2A). Apical and BL cell surfaces were separately treated with PI-PLC, and labeled GPI-APs were captured by the SAE method. The intactness of tight junctions of the polarized monolayer during the PI-PLC treatment has been verified previously by the measurement of protein diffusion across the cell monolayer 35. We identified 29 GPI-APs from the apical surface and 24 GPI-APs from the BL surface (Table2, Supporting Information Tables 7, 8). Protein identifications were highly reproducible with 73% of apically identified GPI-APs and 71% of GPI-APs on the BL surface being observed in two or more biological replicates (Table2, Supporting Information Fig. 1). Notably, 24 of the 29 proteins were observed on both membranes, with only five observed exclusively on the apical surface and no GPI-APs exclusively found on the BL surface (Fig. 2B). While relatively little is known about the polarized distribution of GPI-APs in ARPE-19 cells, the presence of CD73 on the apical and BL membrane domains correlated with its previously reported localization determined by Western blotting, immunofluorescence, and enzymatic activity 35,51. These results successfully demonstrate our ability to identify GPI-APs on discrete membrane domains of live polarized cells.

**Table 2:** Identification of GPI-APs from the apical (AP) and basolateral (BL) surfaces of ARPE-19 cells using sugar analog capture enrichment

No.	Identified proteins	UniProtKB	MWa)	Avg % Seq Cov (Avg Pepb))				Summary	
				AP − PLC	AP + PLC	BL − PLC	BL + PLC	AP	BL
1	5'-Nucleotidase	P21589	63	0	43.3 (19)	8.9 (3)	28.2 (13)	3/3	3/3
2	CD59 glycoprotein	P13987	14	0	20.3 (3)	10.4 (1)	22.4 (3)	3/3	3/3
3	Semaphorin-7A	O75326	75	9.5 (4)	22.1 (11)	10.5 (5)	13.3 (7)	3/3	3/3
4	Glypican-1	P35052	62	9.4 (3)	21.7 (8)	10.2 (4)	14.5 (6)	3/3	3/3
5	Melanotransferrin	P08582	80	0	17.7 (10)	4.4 (2)	12.1 (7)	3/3	3/3
6	Neurotrimin	Q9P121	38	7.5 (1)	18.8 (5)	11.0 (2)	19.9 (5)	3/3	3/3
7	DBH-like monooxygenase protein 1c)	Q6UVY6	70	0	14.4 (7)	2.5 (1)	7.2 (4)	3/3	3/3
8	NKG2D ligand 3	Q9BZM4	28	0	17.6 (3)	0	0	3/3	0/3
9	Reversion-inducing cysteine-rich protein with Kazal motifs	O95980	106	0	17.5 (11)	0	7.5 (6)	3/3	3/3
10	Neuronal growth regulator 1	Q7Z3B1	39	0	16.9 (4)	8.7 (2)	10.3 (3)	3/3	2/3
11	Deoxyribo-nuclease-1-like 1c)	P49184	34	0	19.0 (3)	5.8 (1)	3.9 (1)	2/3	1/3
12	Fibulin-3c)	Q12805	55	16.6 (6)	17.0 (6)	8.7 (3)	10.0 (4)	3/3	3/3
13	Complement decay-accelerating factor	P08174	41	0	13.9 (4)	5.9 (1)	6.6 (3)	3/3	2/3
14	Urokinase plasminogen activator surface receptor	Q03405	37	0	17.2 (4)	6.1 (1)	8.4 (2)	3/3	2/3
15	Lymphocyte function-associated antigen 3	P19256	28	0	8.0 (2)	0	5.1 (1)	2/3	2/3
16	GDNF family receptor alpha-1	P56159	51	0	11.2 (4)	2.2 (1)	5.4 (2)	3/3	2/3
17	CD109 antigen	Q6YHK3	162	4.6 (4)	11.3 (13)	4.3 (4)	5.1 (5)	3/3	2/3
18	Major prion protein	P04156	28	0	6.7 (2)	0	9.0 (2)	2/3	2/3
19	CD44 antigenc)	P16070	82	4.0 (2)	4.0 (2)	4.5 (2)	3.7 (2)	2/3	2/3
20	Netrin-G1	Q9Y2I2	61	0	3.7 (1)	0	0	2/3	0/3
21	Ceruloplasmind)	P00450	122	0.6 (1)	2.3 (2)	0	0	2/3	0/3
22	Glypican-6	Q9Y625	63	0	2.8 (1)	0	0	1/3	0/3
23	Glypican-4	O75487	62	0	5.2 (2)	0	2.5 (1)	1/3	1/3
24	Voltage-gated Ca^2+^ channel subunit α-2/δ-1d)	P54289	125	0	1.5 (1)	0	0.8 (1)	1/3	1/3
25	Acid sphingo-myelinase-like phosphodiesterase 3bc)	Q92485	51	0	3.4 (1)	0	1.6 (1)	1/3	1/3
26	Reticulon-4 receptor-like 2	Q86UN3	46	0	2.7 (1)	0	2.0 (1)	1/3	1/3
27	Carboxypeptidase M	P14384	51	0	1.7 (1)	0	1.4 (1)	1/3	1/3
28	Laminin subunit alpha-4c)	Q16363	203	1.6 (2)	0.8 (1)	0	0	1/3	0/3
29	Contactin-3	Q9P232	113	0	1.2 (1)	0	0.8 (1)	1/3	1/3

The average percent sequence coverage (Avg % Seq Cov) and the average number of unique peptides (Avg Pep) from three biological replicates are given. The reproducibility of a protein identification on a given cell surface in the +PLC samples across three biological replicates is in the far right columns. PLC: phospholipase C, AP: apical, BL: basolateral.

a) Theoretical molecular weight of precursor protein in kDa.

b) The minimum number of unique peptides required for a protein identification was 2, but due to averaging across triplicate samples, the average peptides may appear to be 1. A complete listing of the number of peptides for each replicate can be found in Supporting Information Table 7 and all peptides used in identifications are listed in Supporting Information Table 8.

c) Peptides match specific isoforms predicted to contain a GPI anchor though other isoforms cannot be excluded.

d) Experimentally proven to be GPI anchored in other studies but not annotated as a GPI-AP in the UniProt database.

We next analyzed the GPI proteome of polarized MDCK cells, a cell line that has been extensively used as a model to study polarized GPI-AP trafficking 15,16,18,20,47,52–54. To further improve the sensitivity of our method, 2D LC-MS/MS on a Q Exactive mass spectrometer 55 was employed for the analysis of apical and BL protein samples prepared by SAE. We observed 38 potential GPI-APs from both membranes with 84% of apical protein identifications and 95% of BL protein identifications observed in two or more biological replicates (Table3, Supporting Information Fig. 1). Detailed information on all the identified GPI-APs in the biological triplicate samples including spectra counts and peptide assignments can be found in Supporting Information Tables 9 and 10. Notably, while MDCK GPI-APs are thought to be preferentially trafficked to the apical surface, we observed most detected GPI-APs on both cell surfaces (35 out of 38, or 92%), with only one GPI-AP being exclusively detected on the apical surface, and two GPI-APs present only on the BL surface (Fig. 2C; Table3). Importantly, we identified carboxypeptidase M, a GPI-AP known to be present on both the apical and BL membranes of MDCK cells 47 (Table3). Together, these data demonstrate that GPI-APs are clearly present on both surfaces of polarized epithelial cells.

**Table 3:** Identification of GPI-APs from the apical (AP) and basolateral (BL) surfaces of MDCK cells using sugar analog enrichment

No.	Identified protein	Accession	MWa)	Avg % Seq Cov (Avg Pepb)				Summary	
				AP − PLC	AP + PLC	BL − PLC	BL + PLC	AP	BL
1	Folate receptor beta	XP_534020.3, XP_005633610.1	31	36.4 (7)	53.3 (12)	22.1 (5)	54.2 (14)	3/3	3/3
2	Limbic system-associated membrane protein	XP_003434117.1, XP_005639572.1	40	36.2 (12)	36.9 (12)	22.2 (6)	41.6 (14)	3/3	3/3
3	Carboxypeptidase M	XP_005625715.1	60	18.1 (8)	28.0 (15)	8.7 (4)	29.7 (16)	3/3	3/3
4	CD59 glycoprotein	XP_533156.1, XP_005631182.1, XP_005631183.1, XP_005631184.1, XP_005631185.1, XP_005631186.1, XP_005631187.1	14	6.8 (1)	26.0 (5)	6.8 (1)	24.1 (4)	3/3	3/3
5	CD109 antigen	XP_532205.3	162	36.5 (41)	48.2 (58)	27.0 (30)	50.6 (62)	3/3	3/3
6	Pantetheinase precursor	NP_001003372.1	57	10.6 (5)	31.1 (14)	8.6 (4)	40.1 (18)	3/3	3/3
7	ADP-ribosyl cyclase 2	XP_545938.2	35	16.2 (4)	46.6 (10)	9.6 (2)	47.6 (12)	3/3	3/3
8	Ceruloplasmin	XP_005634613.1, XP_534301.2	126	26.0 (18)	39.2 (33)	28.9 (20)	42.1 (36)	3/3	3/3
9	Deoxyribonuclease I-like 1	XP_005642080.1	34	11.3 (2)	40.4 (9)	6.9 (2)	46.1 (13)	3/3	3/3
10	Hyaluronidase-2	XP_541876.2, XP_005632567.1, XP_005632568.1, XP_005632569.1, XP_005632570.1	53	5.9 (2)	15.6 (7)	7.9 (3)	27.0 (12)	3/3	3/3
11	Folate receptor alpha	XP_005633611.1, XP_851993.1	29	0	5.3 (1)	0	19.2 (4)	1/3	3/3
12	Urokinase plasminogen activator surface receptor	XP_003432622.3	40	27.3 (7)	29.1 (7)	12.5 (3)	29.1 (7)	3/3	3/3
13	Glypican-1	XP_005636000.1	67	26.5 (12)	32.9 (16)	29.0 (14)	35.9 (18)	3/3	3/3
14	Dipeptidase 1	XP_536748.3	50	1.3 (1)	20.7 (7)	0	42.3 (15)	3/3	3/3
15	Glypican-4	XP_549265.2	62	10.0 (4)	21.1 (9)	8.9 (3)	39.7 (18)	3/3	3/3
16	Ephrin-A1	XP_547553.1, XP_852071.1, XP_005622810.1, XP_005622811.1, XP_005622812.1, XP_005622813.1,	24	18.8 (3)	13.9 (2)	18.2 (2)	36.2 (4)	2/3	3/3
17	Melanotransferrin	XP_005639711.1	87	8.4 (5)	37.3 (23)	10.4 (6)	43.2 (28)	3/3	3/3
18	Alkaline phosphatase, tissue-nonspecific isozyme	XP_005617269.1, XP_005617270.1, XP_005617271.1, NP_001184066.1	58	3.2 (1)	22.5 (10)	6.2 (3)	35.4 (15)	3/3	3/3
19	Major prion protein precursor	NP_001013441.1	28	4.3 (1)	18.5 (3)	0	24.0 (4)	3/3	3/3
20	Mesothelin	XP_854112.1	45	17.0 (5)	15.0 (4)	3.4 (1)	25.4 (7)	3/3	3/3
21	Semaphorin-7A	XP_005638673.1	73	16.7 (9)	22.9 (12)	19.4 (10)	32.2 (16)	3/3	3/3
22	Leishmanolysin-like peptidase	XP_851508.3	81	4.4 (3)	17.0 (11)	0.9 (1)	23.2 (15)	3/3	3/3
23	Monocyte differentiation antigen CD14	XP_848746.2	40	7.5 (3)	22.1 (6)	8.8 (3)	32.4 (9)	3/3	3/3
24	Protein APCDD1	XP_537333.2	59	0	1.9 (1)	0	33.3 (13)	1/3	3/3
25	Acid sphingomyelinase-like phosphodiesterase 3b	XP_005617767.1	51	0	9.7 (3)	3.0 (1)	32.8 (11)	2/3	3/3
26	Bone marrow stromal antigen 2	XP_865603.1	21	11.9 (2)	15.8 (3)	0	21.3 (4)	3/3	3/3
27	Ly6/PLAUR domain-containing protein 6B	XP_005632045.1	18	0	0	0	11.3 (1)	0/3	2/3
28	Growth arrest-specific protein 1	XP_849279.1	35	0	8.7 (2)	0	7.3 (2)	3/3	3/3
29	Matrix metalloproteinase-17	XP_852332.3	72	0	1.8 (1)	0	6.0 (3)	1/3	3/3
30	Reversion-inducing cysteine-rich protein with Kazal motifs	XP_005626418.1, XP_005626417.1, NP_001002985.1, XP_005626419.1,	110	0.6 (1)	1.6 (1)	0	2.2 (2)	1/3	2/3
31	Prostasin	XP_005621300.1, XP_005621299.1	36	2.8 (1)	10.5 (2)	0	21.0 (3)	3/3	3/3
32	NKG2D ligand 1	XP_003432589.2	29	30.9 (9)	39.3 (11)	18.0 (5)	42.5 (13)	3/3	3/3
33	Complement decay-accelerating factor	XP_005622370.1, XP_005622369.1, XP_005622371.1, XP_005622372.1	71	5.4 (3)	19.5 (11)	6.4 (3)	19.3 (12)	3/3	3/3
34	Lipoprotein lipase	XP_005635791.1, XP_005635790.1	53	4.2 (1)	7.4 (2)	6.2 (2)	6.2 (2)	2/3	2/3
35	EGF containing fibulin-like extracellular matrix protein 1	XP_531834.1, XP_005626175.1, XP_005626176.1	55	18.5 (7)	23.1 (8)	20.1 (8)	23.5 (9)	3/3	3/3
36	Pantetheinase-like	XP_005615740.1	44	0	4.4 (1)	0	0	1/3	0/3
37	MAM domain-containing GPI anchor protein 1	XP_532128.3	106	0	0.6 (1)	0	1.5 (1)	1/3	1/3
38	Trehalase	XP_005619766.1	66	0	0	0	1.5 (1)	0/3	1/3

The average percent sequence coverage (Avg % Seq Cov) and average number of unique peptides (Avg Pep) across three biological replicates are indicated. The reproducibility of a protein identification on a given cell surface in the +PLC samples across three biological replicates is in the far right columns. PLC: phospholipase C, AP: apical, BL: basolateral.

a) Predicted molecular weight in kDa. When more than one isoform is listed, the predicted molecular weight of the largest isoform is given.

b) The minimum number of unique peptides required for a protein identification was 2, but due to averaging across triplicate samples, the average peptides may appear to be 1. A complete listing of the number of peptides for each replicate can be found in Supporting Information Table 9 and all peptides used in identifications are listed in Supporting Information Table 10.

## 4 Discussion

We report methodology for identification of GPI-APs directly from the surface of intact mammalian cells using bottom-up proteomics. An enabling feature of our experimental approach was the use of a sample preparation method that selectively released GPI-APs (using PI-PLC) from the surface of intact cells coupled to the capture and enrichment of GPI-APs via their ubiquitous appended glycans. Using these methods upstream of LC-MS/MS, we have analyzed the cell surface GPI proteomes of three mammalian cells lines with significantly improved depth (see Supporting Information Table 11 for a summary of all identified GPI-APs across the three cell lines). Furthermore, the ability to identify cell surface GPI-APs without perturbing cellular membranes has permitted us to separately define the GPI proteomes of distinct plasma membrane domains of polarized epithelial cells.

### 4.1 Considerations in the choice of GPI-AP enrichment strategy

Variations in the abundance and/or composition of glycans on individual GPI-APs are expected to play a role in the sensitivity of the glycan enrichment method. Glycan-rich proteins may perform well in both enrichment schemes due to an increased likelihood of GalNAz incorporation or association with the lectin resin. In contrast, GPI-APs bearing fewer glycans and/or glycans that lack epitopes recognized by WGA or ConA may be more difficult to detect. However, in our HeLa samples, we do identify proteins with a single predicted site of N- or O-glycosylation, suggesting our method can detect GPI-APs even if they are not heavily glycosylated. Additionally, GPI-APs that have tertiary folds or other modifications that sterically hinder the ability of a glycan to interact with the capture resin may favor the identification by only one method. In support of this, we readily detected the heparan sulfate modified glypican family of GPI-APs (glypican 1, 4, and 5) 56 via sugar analog capture enrichment but not with lectin affinity capture enrichment. We speculate that heparan sulfate may sterically interfere with glycan interaction with the lectins used in this study. The performance of lectin affinity capture enrichment in this workflow may be improved by including additional immobilized lectins in the resin mixture whose specificities widen the array of recognized glycan epitopes 57 and/or through the use of lectin multimerization 58.

Unique features of the enrichment methods indicate their suitability for a particular application. We generally observed a greater number of identified GPI-APs with sugar analog capture enrichment than with lectin affinity capture enrichment, likely in part due to the covalent interaction between the incorporated sugar and the agarose resin. However, we have seen differences in the efficiency of GalNAz incorporation into glycans in different cell lines, thus necessitating optimization of labeling conditions for each cell type. In contrast, lectin affinity capture enrichment may be advantageous because metabolic labeling of glycans is not necessary to isolate GPI-APs and its specificity can be tuned to specific glycan epitopes. This method could be applied to the analysis of GPI-APs from cells or tissues directly extracted from humans or other animals. Furthermore, due to the noncovalent nature of the lectin-GPI-AP interaction, eluted GPI-APs could be further analyzed to determine the structure of GPI-linked glycans, a type of analysis that would not be possible using sugar analog capture because the glycans become irreversibly bound to the alkyne agarose column. Finally, lectin capture may also permit identification of proteins that specifically interact with GPI-APs.

Lastly, while both glycan enrichment methods are effective in capturing GPI-APs after PI-PLC release from the cell surface, factors that potentially limit the efficiency of PI-PLC digestion could cause some GPI-APs to be missed. It has been shown previously that residual carboxypeptidase M activity remains in the apical membrane of MDCK cells after PI-PLC treatment 47. This suggests that the apical surface of MDCK cells may be less accessible to PI-PLC or that a subset of GPI-APs on this surface are modified thus rendering them resistant to PI-PLC cleavage. Consistent with the latter, resistance to PI-PLC has been observed previously in erythrocytes and is due to acylation of the GPI inositol 59,60. We did observe less enrichment upon PI-PLC treatment in the apical samples (1.4-fold) as compared to the BL surface (3.0-fold; Supporting Information Table 6) suggesting that perhaps some apical GPI-APs are resistant to PI-PLC cleavage. Oligomerization of GPI-APs during trafficking to the cell surface 52 or clustering of GPI-APs in lipid rafts 61 could also potentially limit access of PI-PLC to their GPIs. However, we identified 17 of the 19 GPI-APs previously found in the HeLa cell lipid raft proteome 33, suggesting that in that cell line, PI-PLC cleavage is relatively efficient and complete despite clustering of GPI-APs in lipid rafts.

### 4.2 GPI-APs in polarized epithelial cells

Prior to this study, little was known about the cohort of GPI-APs that naturally populates the surface of polarized mammalian epithelial cells. We have advanced this knowledge by cataloging the GPI-APs present on the apical and BL surfaces of both ARPE-19 and MDCK cells. We identified a large number of GPI-APs having diverse molecular functions, the majority of which were not previously known to be produced in these cell lines. Several studies have indicated that GPI-APs are preferentially trafficked to the apical surface of polarized ARPE-19 and MDCK cells 15,17,20,51. Based on these data, one would anticipate that most GPI-APs would be present predominantly in apically derived samples. However, GPI-APs were detected on both the apical and BL surfaces, with very few being present on only one membrane domain. This could be due to our use of sensitive MS technology to detect endogenous GPI-APs in comparison with previous reports that used exogenous reporter GPI-APs or activity assays. Importantly, while GPI-APs are present on both membrane domains of polarized cells, it remains to be determined if these proteins are active in both domains. The increased knowledge we now have of the endogenous GPI-APs that populate these membranes will permit a more rigorous exploration of their individual localization, activity, and surface abundance.

### 4.3 Future applications

We have developed two new methods to enrich and identify GPI-APs from the complex mixture of proteins present on the surface of living cells. Importantly, these approaches permit exploration of the mammalian GPI-anchored proteome without disturbing cellular integrity. We anticipate that these methods will further enable novel biomarker discovery, the monitoring of changes in the GPI proteome during cell differentiation or disease progression, and in-depth characterization of the glycan moieties appended to GPI-APs.

## Abbreviations

AP: apical
ARPE-19: human retinal pigment epithelium cells
BL: basolateral
ConA: Concanavalin A
FDR: false discovery rate
GalNAz: *N*-azidoacetylgalactosamine
GlcNAc: *N*-acetylglucosamine
GPI-AP: glycosylphosphatidylinositol-anchored protein
MDCK: Madin–Darby canine kidney cells
PI-PLC or PLC: phosphatidylinositol-specific phospholipase C
SAE: sugar analog enrichment
WGA: wheat germ agglutinin
