# Supplementary material for: Proteomic identification of mammalian cell surface derived glycosylphosphatidylinositol-anchored proteins through selective glycan enrichment
Source: Proteomics. 2014 Nov 12;14(21-22):2471–84. doi: 10.1002/pmic.201400148 (PMC4260145; doi:10.1002/pmic.201400148)
Supplement: Supplementary file 12 [file pmic0014-2471-SD12.doc]

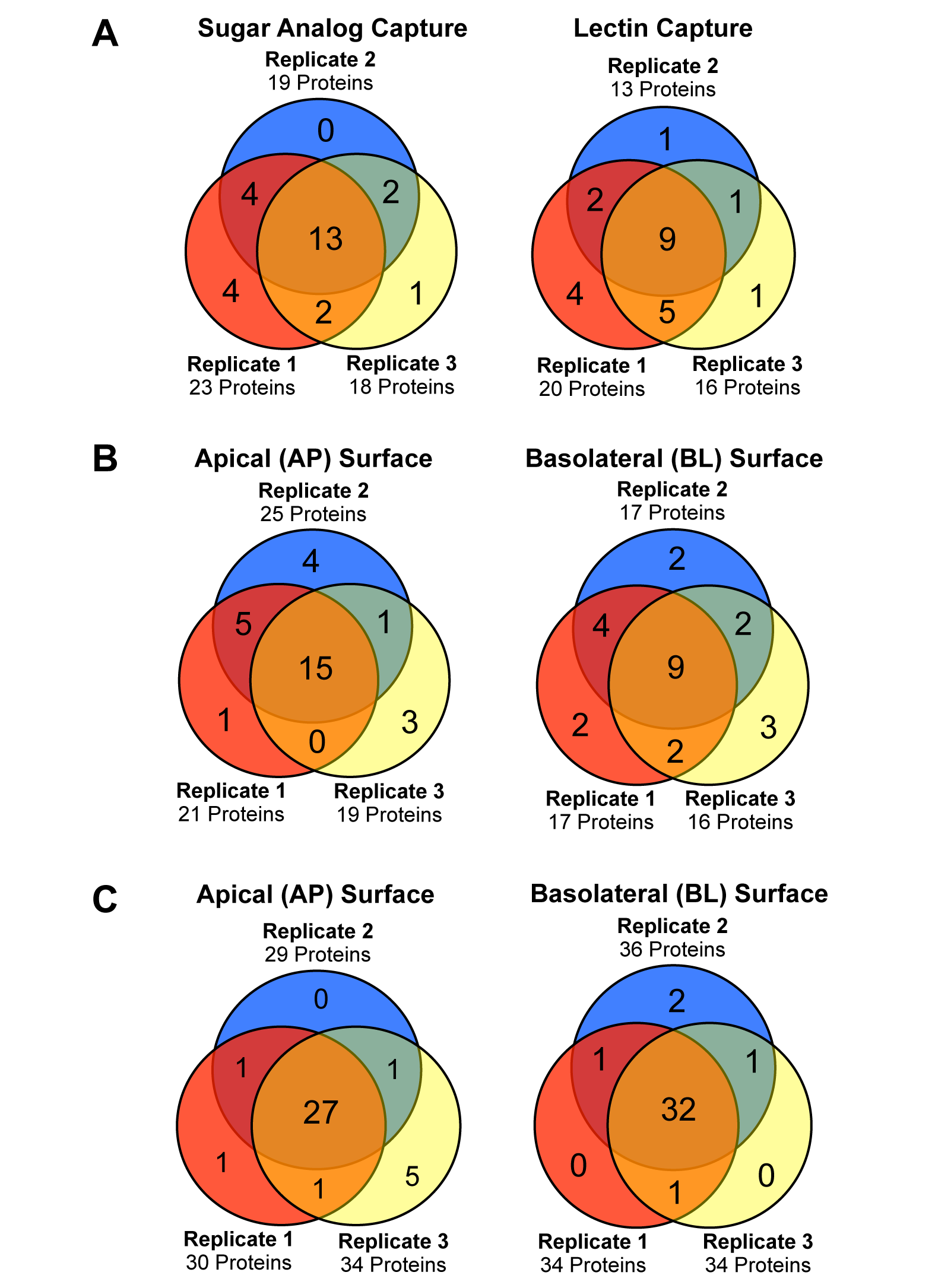


**Supplementary Figure S1. Reproducibility of GPI-AP identification across biological triplicate experiments.** A. HeLa cells, using sugar analog capture enrichment (left) and lectin affinity capture enrichment (right). B. Polarized ARPE-19 cells using sugar analog capture enrichment on GPI-APs liberated from the apical (left) and basolateral (right) surfaces of the cells. C. Polarized MDCK II cells using sugar analog capture enrichment on the apical (left) and basolateral (right) surfaces of cells.
